# A New Antiseptic in Dentistry

**Published:** 1891-04

**Authors:** W. H. Whitslar


### A New Antiseptic in Dentistry.

W. H. WHITSLAR, D.D.S.

In the January number of the REGISTER attention was called to *Aristol*, a new antiseptic introduced to take the place of iodoform, iodole and sozo-iodol. It now transpires that aristol is making its way into use by the dental profession on account of its merits. Like many new drugs which have come highly recommended by the manufacturers, aristol receives its quota of advertisement, but it will be noticed that testimonials for its worth are from men of noted rank in medical lore. Now in the dental field of labor, Dr.

J. V. Kejzlar of Jicin, in the *Austro-Hungarian Dental Quarterly*, October, 1890, reports his use of aristol, which is as follows:

"Some time since, my attention was drawn to a new, powerful antiseptic, the thymol derivative called Aristol, discovered by Drs. Messinger and Vortmann, of Aachen.

It is the best substitute for Iodoform, which, on account of its disgusting odor, is especially abhored by patients who have disorders of the mouth or teeth. Aristol has no disagreeable odor and is easily endured by sensitive patients. It has no toxic properties like Iodoform, does not irritate, adheres very readily to loose-lying pulps, and has excellent healing properties.

I tried it in all cases of the most varied tooth complaints, in which the hitherto available antiseptics had been used, such as gangrenous pulps, antiseptic for the root canals, disinfection of carious cavities, before introducing the filling, and so on.

On gangrenous pulps, I strewed Aristol in powder with a fine brush; for disinfecting the root canals and carious cavities, I used a ten per cent. solution of Aristol in sulphuric ether; through the rapid evaporation of the ether, the Aristol forms an even coating and rapidly dries the cavity.

For fistulous spaces, I use small rods made of ten parts cocoa butter and one part Aristol, by which granulation and healing proceed rapidly. Aristol is insoluble in water, but readily soluble in sulphuric ether, collodion and fatty substances. It is generally used in a ten per cent. solution or directly in the powdered form, externally for wounds, all kinds of swellings, for eruptions, syphilis, ozæna, ottorhœa nasofaryngal syphilis, in gynækology and wherever antiseptics are indicated for external use.

Aristol may be regarded as an enrichment of materia medica, especially for disorders of the teeth, and it is very desirable that the remedy be further tried in this direction."

Supplementing the above it may be added that in the filling of root canals, whether results are to be credited to aristol alone, or to pure cleanliness, or to all the steps in the operation it is sometimes hard to judge, but we have been pleased to notice a less number of cases where apical inflammation was reported since the use of aristol was begun. Out of the ninety (90) root fillings made, the following result was noted : in one case (lateral incisor), root filling was packed too tight, and the patient allowed suppuration to supervene; in another case, inferior third molar, rubber dam could not be adjusted, there resulted considerable inflammation which of its own accord subsided in a few days ; only two other cases were reported of any inflammation whatever. Without entering into a discussion of root filling, we maintain that in some cases only absolute cleanliness may be necessary preparatory to root filling and no antiseptic dressing is required. The majority of cases however do need a dressing and we have found in our hands, aristol to be invaluable. It is useful in ulitis, and in painful ulcerations of the mouth. We have used it in ulcerations of the cervix uteri with success. Dissolved in chloroform about sixty grains to one-half ounce it makes a dressing that is alike useful in pumping into root canals of teeth, or covering an excoriation of the skin ; in each it forms an impervious coating almost like varnish, and moisture will not ooze through it. Thus will be seen its value in root filling. The apical foramen of the tooth is sealed as also are the tubuli. But we do not wish to go wild over a new thing. Let time and trials prove its worth. All things must seek their level.

